# The predictive value of left atrioventricular coupling index for ILR-detected atrial fibrillation following an embolic stroke of undetermined source in patients with sinus rhythm

**DOI:** 10.1093/ehjimp/qyag065

**Published:** 2026-04-09

**Authors:** Rahul K Chattopadhyay, Panagiota A Chousou, Navazh Jalaludeen, Gareth Matthews, Liam Ring, Joseph Cheriyan, Peter J Pugh, Vassilios S Vassiliou

**Affiliations:** Department of Cardiac Medicine, Norwich Medical School, University of East Anglia, Norwich Research Park, Norwich, Norfolk NR4 7TJ, UK; Department of Cardiology, Addenbrookes Hospital, Cambridge University Hospitals NHS Foundation Trust, Hill’s Road, Cambridge CB2 0QQ, UK; Department of Clinical Pharmacology, Cambridge University Hospitals NHS Foundation Trust, Hill’s Road, Cambridge CB2 0QQ, UK; Department of Cardiac Medicine, Norwich Medical School, University of East Anglia, Norwich Research Park, Norwich, Norfolk NR4 7TJ, UK; Department of Cardiology, West Suffolk Hospital, Hardwick Lane, Bury St Edmunds, Bury Saint Edmunds IP33 2QZ, UK; Department of Clinical Pharmacology, Cambridge University Hospitals NHS Foundation Trust, Hill’s Road, Cambridge CB2 0QQ, UK; Department of Cardiology, Addenbrookes Hospital, Cambridge University Hospitals NHS Foundation Trust, Hill’s Road, Cambridge CB2 0QQ, UK; Department of Cardiac Medicine, Norwich Medical School, University of East Anglia, Norwich Research Park, Norwich, Norfolk NR4 7TJ, UK

**Keywords:** atrial fibrillation, left atrioventricular coupling index, embolic stroke of undetermined source, implantable loop recorder

## Abstract

The prediction of atrial fibrillation in high-risk populations is an important research area of modern atrial fibrillation care. One such population is the embolic stroke of undetermined source population, where appropriate anticoagulation might help reduce the annual stroke recurrence risk by 5%. In this study, individuals were categorized according to their tertile of left atrioventricular coupling index, a composite echocardiographic parameter that reflects both left atrial and ventricular size. Its association with the detection of an implantable loop recorder and subclinical atrial fibrillation was investigated. A retrospective single-centre cohort study was performed. A total of 296 embolic stroke of undetermined source patients, referred for an implantable loop recorder, who also underwent echocardiography, were screened. Of these, 230 cases had echocardiograms of sufficient quality to measure the left atrioventricular coupling index. Patients were categorized according to their left atrioventricular coupling index tertile, with tertile 1 being less than 16.97, tertile 2 being 16.97–23.9, and tertile 3 being greater than 23.9. Patients in the third left atrioventricular coupling tertile were more likely to develop atrial fibrillation of any duration, with a 1.9-fold increased univariable hazard ratio, although this association was not significant in longer duration of atrial fibrillation episode. This effect was no longer significant with stepwise multivariable analysis, suggesting that age was primarily mediating the effect seen in this study. Left atrioventricular coupling index was not an independent predictor of future implantable loop recorder-detected atrial fibrillation after adjustment for age in this cohort.

## Graphical Abstract

**Graphical Abstract:**
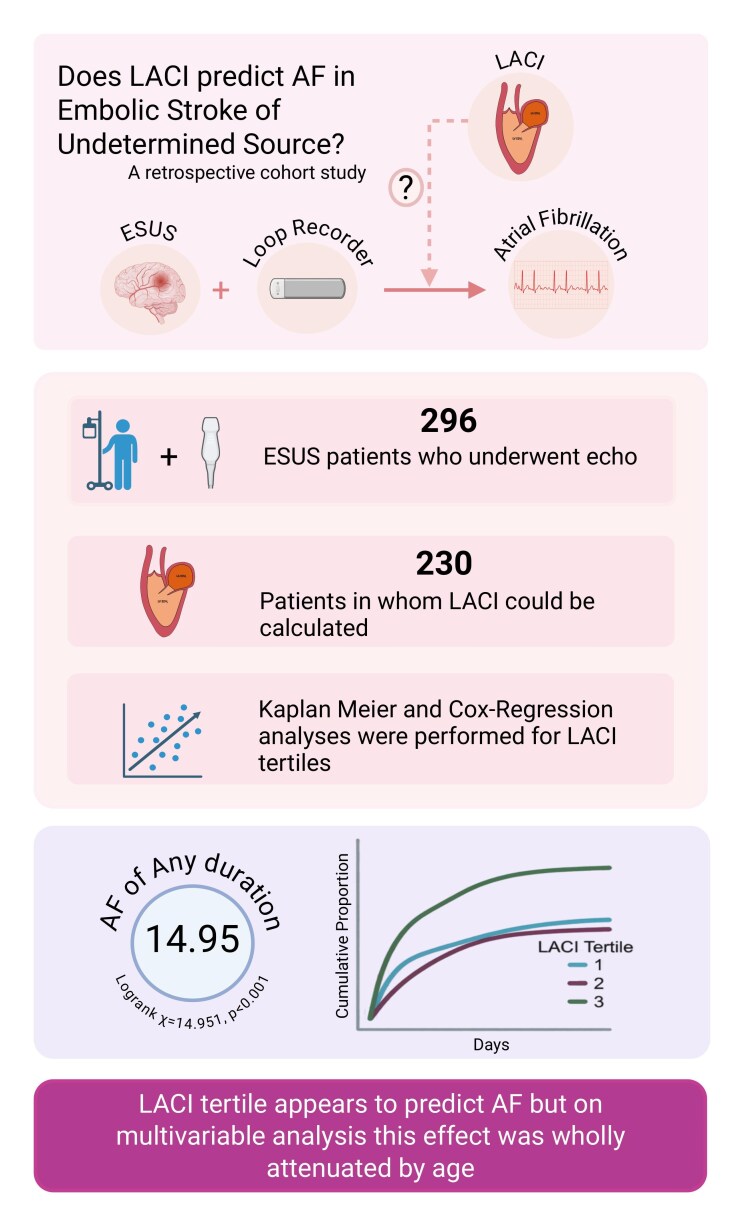

## Introduction

The prediction of atrial fibrillation (AF) in patients with sinus rhythm is an area of growing research interest. This is particularly true in the embolic stroke of undetermined source (ESUS) population, where implantable loop recorder (ILR) detected AF rates have been reported to be as high as 48.6%,^1^ and the detection of AF prompts consideration of anticoagulation therapy. ILR-detected AF is generally considered subclinical AF (SCAF) in the absence of symptoms or prolonged durations.

There is increased appreciation that the interplay between the ventricle and atria plays an important role in AF pathogenesis. The left atrioventricular coupling index (LACI) is a parameter that relates left atrial and ventricular volumes, and in doing so is an integrative marker of both left atrial and left ventricular physiology.^2^ LACI is defined as the ratio of the indexed left atrial volume at ventricular end-diastole (when the left atrium is at its minimal volume) and the indexed left ventricular end-diastolic volume.^3^

Recently, a study highlighted the predictive role of LACI in heart failure, with individuals in the lowest tertile of LACI being most likely to be hospitalized or die.^4^ This analysis, therefore, aimed to explore whether categorization into LACI tertiles may be a useful predictor of future ILR-detected SCAF in a high-risk ESUS population.

## Methods

### Study design

The original study methodology has previously been reported.^5^ In brief, a retrospective analysis was performed on ESUS patients who were referred by their stroke physician for AF monitoring by ILR implantation.

This was a single-centre study, including a total of 296 ESUS patients identified between December 2009 and September 2019, who had undergone echocardiography. This observational study was approved by the United Kingdom Health Research Authority (16/NW/0527). Institutional approval was given by Cambridge University Hospitals NHS Foundation Trust. The North West Preston Research Ethics committee waived the need for signed consent in view of the retrospective nature of the study. The study is registered at ClinicalTrials.gov (NCT02843516) and complies with the 1975 Declaration of Helsinki for research. Transthoracic echocardiograms were performed for patients while in sinus rhythm, with offline analysis (EchoPAC v203.59, GE) performed by a British Society of Echocardiography accredited cardiologist.

The primary outcome was the correlation of LACI tertile with the subsequent detection of subclinical AF detected on the ILR across differing durations: of any duration, >6 min duration, and >5.5 h. All ILR algorithm-detected and patient-triggered episodes were reviewed by two European Heart Rhythm Association–accredited cardiologists with a special interest in arrhythmia and devices, with a third cardiologist reviewing in cases of disagreement. AF was defined as an irregular heart rhythm, with no visible *P*-waves. For the purposes of this analysis, atrial flutter episodes were considered synonymous with AF.

### Left atrioventricular coupling index

LACI measurements were performed at the same end-diastolic phase, defined by the frame before mitral valve closure, in the same cardiac cycle. The cohort was ordered according to LACI value, and then, three tertiles of equal size were created, with tertile 3 representing the highest LACI values. Higher LACI values represent a larger left atrial to left ventricular end-diastolic volume, thus reflecting worse coupling of the two chambers.

### Statistical analysis

Statistical analysis was performed used IBM SPSS Statistics (version 31). The population was divided into tertiles according to the LACI. Continuous variables were presented as mean ± standard deviation and compared between groups using the unpaired one-way ANOVA test. Categorical variables were compared by the χ2 test with Yates correction or the Fisher exact test. When a significant difference was noted, a Tukey *post hoc* analysis was performed to identify which groups were different. Kaplan-Meier survival analyses were conducted to compare the cumulative incidence of AF occurrence according to LACI tertiles. The log-rank test was used to test whether there was a difference between the Kaplan-Meier curves. Cox proportional hazards regression was used to calculate the hazard ratios and 95% confidence intervals for the risk of AF development according to LACI tertile. Univariable predictors were included in the multivariable analysis. Established predictors of SCAF were treated as covariates for the purposes of multivariable analysis.

## Results

It was possible to calculate the left atrioventricular coupling index in a total of 230 patients from the cohort. The average age of the cohort was 55.4 years (SD 14.8 years), with a total of 85 women (36.8%). Mean follow- up time was 822 days (SD 615 days). *Table 1* summarizes the patient characteristics according to LACI tertile.

**Table 1:** Baseline population characteristics

	All	LACI Tertile 1	LACI Tertile 2	LACI Tertile 3	*P*
	*n* = 230	*n* = 76	*n* = 77	*n* = 77	
Age (years)	55.4 (14.8)	49.9 (13.3)	53.8 (15.0)	62.3 (13.2)	<0.001^a,b^
BMI (kg/m^2^)	27.4 (4.5)	26.0 (4.5)	28.2 (4.2)	28.1 (4.6)	0.004^a,c^
Female (%)	85	27	23	35	0.128
Diastolic blood pressure (mmHg)	75.7 (10.6)	76.4 (10.2)	74.4 (10.3)	76.4 (11.4)	0.403
Systolic blood pressure (mmHg)	130.5 (18.6)	128.7 (17.3)	129.4 (18.2)	133.2 (19.8)	0.269
Time to AF detection (days)	883.3 (595.4)	883.3 (595.4)	922.3 (560.0)	597.0 (586.2)	<0.01^a,c^
PFO (%)	84 (36.5)	36 (47.4)	30 (39.0)	18 (23.4)	0.007^b,c^
Heart failure (%)	1 (0.4)	0 (0)	0 (0)	1 (1.3)	0.369
Hypertension (%)	96 (41.7)	29 (38.2)	29 (37.7)	38 (49.4)	0.251
Diabetes (%)	27 (11.7)	10 (13.2)	9 (11.7)	8 (10.4)	0.868
Vascular disease^d^ (%)	91 (39.6)	27 (35.5)	27 (35.1)	37 (48.1)	0.175
*AF*					
Any duration (%)	123 (53.5)	35 (46.1)	35 (45.5)	53 (68.8)	0.004^b,c^
AF > 6 min (%)	35 (15.2)	12 (15.8)	8 (10.4)	15 (19.5)	0.287
AF > 5.5 h (%)	19 (8.3)	7 (9.2)	4 (5.2)	8 (10.4)	0.471
*Echocardiographic parameters*					
LA diameter (cm)	3.39 (0.54)	3.21 (0.49)	3.44 (0.49)	3.52 (0.58)	<0.001^a,c^
LVEDVi (mL/m^2^)	55.4 (12.7)	58.4 (12.4)	56.3 (12.8)	51.6 (12.0)	0.003^c^
LVESVi (mL/m^2^)	21.9 (7.07)	22.9 (7.3)	21.5 (5.4)	21.2 (8.2)	0.283
LV SV (mL)	65.7 (17.7)	68.9 (17.1)	69.2 (18.5)	59.1 (15.7)	<0.001^a,c^
LV EF (%)	60.8 (5.8)	61.1 (6.3)	61.8 (4.4)	59.4 (6.5)	0.036^b^
Lateral PA (s)	75.3 (20.0)	76.6 (19.0)	71.2 (19.7)	78.2 (20.9)	0.104
LAV_min I_ (mL/m^2^)	11.7 (5.1)	8.08 (2.33)	11.1 (2.71)	15.8 (5.94)	<0.001^a,b,c^
LA emptying fraction (%)	56.3 (8.0)	61.2 (6.74)	56.9 (5.41)	50.8 (7.86)	<0.001^a,b,c^
LARS (%)	27.1 (8.9)	32.0 (9.51)	27.6 (7.30)	21.7 (6.4)	<0.001^a,b,c^
Atrial septal aneurysm (%)	21 (9.1)	7 (9.2)	5 (6.5)	9 (11.7)	0.534

Continuous variables are represented by mean and standard deviation. Categorical variables are represented by the count and percentage. A one-way analysis of variance was performed for normally distributed continuous variables, a χ^2^ test was used for categorical variables.

AF, atrial fibrillation; BMI, body mass index; kg, kilogram, LACI, left atrioventricular coupling index; LASR, left atrial reservoir strain; lateral PA, time interval from the beginning of the *P* wave on the surface electrocardiogram to the beginning of the A′ wave on pulsed wave tissue Doppler of the lateral mitral annulus; LVEDVi, indexed left ventricular end-diastolic volume; LVEF, left ventricular ejection fraction; m, metres; ml, millilitre; mmHg, millimetres of Mercury; PFO, patent foramen ovale; SD, standard deviation.

^a^Demonstrates statistically significant differences (*P* < 0.05) between tertiles 1 and 3.

^b^Demonstrates statistically significant differences (*P* < 0.05) between tertiles 2 and 3.

^c^Demonstrates statistically significant differences (*P* < 0.05) between tertiles 1 and 2.

^d^Defined by the presence of previous myocardial infarction or coronary artery disease, peripheral vascular disease or aortic plaque.

Over the course of the follow-up, AF was detected in a total of 123 patients (53.2%), with the proportion of patients developing AF in the worst tertile being considerably higher than the other tertiles (46.0% in tertile 1; 45.5% in tertile 2% and 68.8% in tertile 3, *P* = 0.04). The yield of longer durations of AF was significantly smaller, with only 35 patients (15.2%) developing AF of over 6 min and 19 patients (8.3%) developing AF of over 5.5 h. AF detection was identified earlier in the worst LACI tertile [tertile 1–883.3 days (SD 595.4 days), tertile 2–922.3 days (SD 560.0 days), tertile 3 −597.0 days (SD 586.2 days), *P* < 0.01].

When echocardiographic characteristics are considered, there is an increase in LA size and a reduction in the indexed LV end-diastolic volume in LACI tertile 3 compared to tertiles 1 and 2. There is also a reduction in measures of LA function in each LACI tertile, with both the LA emptying fraction and the LA reservoir strain reducing as the LACI tertile worsened.

**Figure 1:**
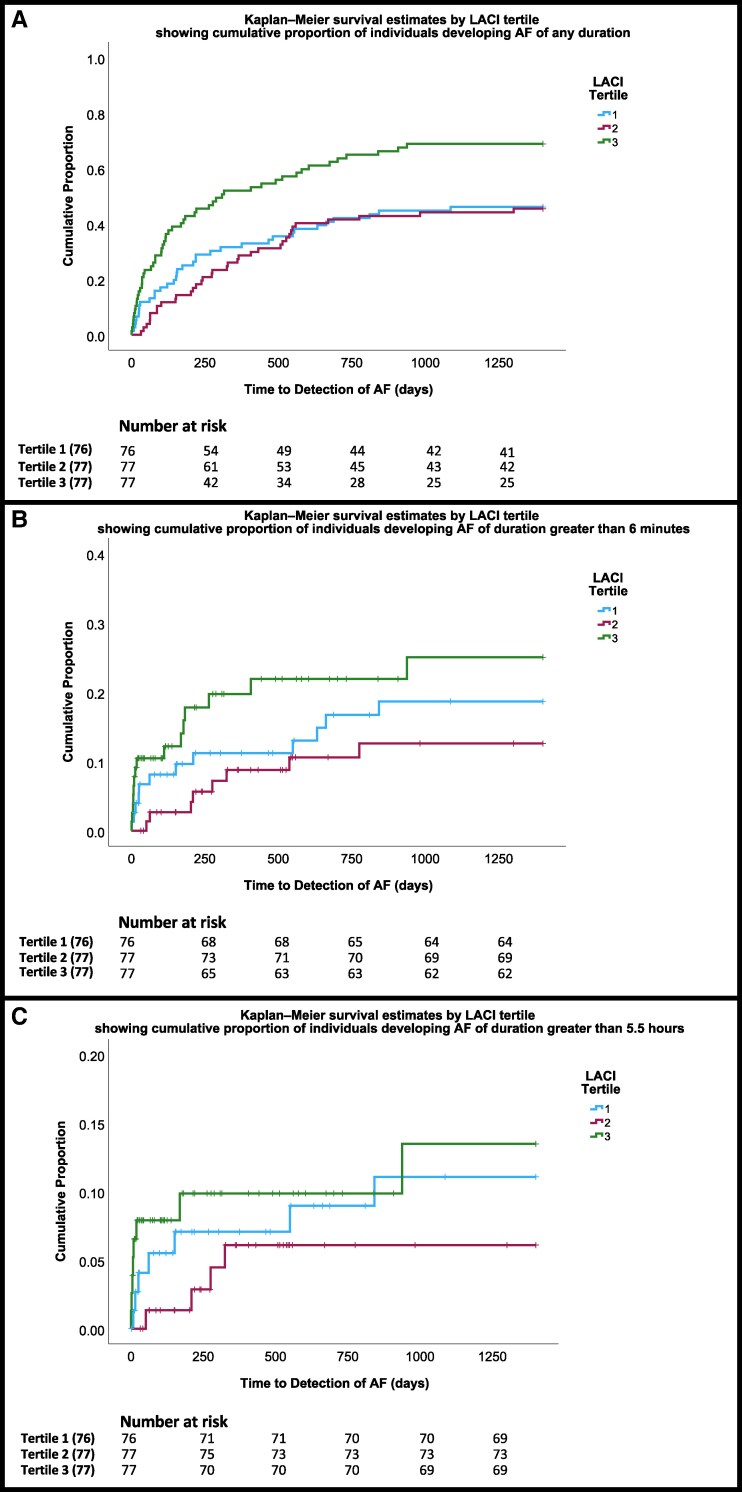
(A–C) Kaplan-Meier curves showing the association between LACI tertile and the cumulative occurrence of AF. The number of patients who were at risk of AF development at entry into each time point has been included in a life table. Top panel (*A*) Any duration of AF, middle panel (*B*) Over 6 min of AF, bottom panel (*C*) Over 5.5 h of AF.

Kaplan-Meier survival analysis curves are shown in *Figure 1*. For any duration of AF, the log-rank value was χ2 = 14.951, *P* < 0.001; for greater than 6 min of AF, the log-rank value was χ2 = 4.459, *P* = 0.108; and for over 5.5 h of AF, the log-rank value was χ2 = 2.408, *P* = 0.300.

A Cox proportional regression model was performed to further investigate the predictive role of LACI tertile for the detection of any duration of SCAF. The proportional hazards assumption was checked, and no violations were detected, supporting the validity of the model. Univariable analyses were performed first, with LACI tertiles and other known predictors of AF (Table 2). Compared to patients in the first tertile of LACI, the worst tertile is associated with a univariable 1.9-fold increased hazard of AF development. After multivariable adjustment, the only predictor that remained significant was increasing age, with stepwise analysis suggesting that the univariable association between the worst LACI tertile and AF occurrence is mediated by increasing age (Table 2).

**Table 2:** Univariate and multivariate Cox regression analyses

			Univariate		Multivariate	
	Total Number	Number who developed AF (%)	HR (95% CI)	*P*-value	HR (95% CI)	*P*-value
Female gender	85	49 (57.6)	1.232 (0.859–1.768)	0.257		
Age	230	112 (48.7)	1.032 (1.019–1.045)	**<0**.**001**	1.030 (1.013–1.047)	**<0**.**001**
BMI	230	112(48.7)	0.970 (0.932–1.010)	0.97		
Diastolic blood pressure	228	111 (48.3)	1.023 (1.006–1.041)	**0**.**009**	1.023 (0.999–1.047)	0.063
Systolic blood pressure	228	111(48.3)	1.011 (1.002–1.020)	**0**.**018**	0.994 (0.981–1.007)	0.370
Diabetes	27	14 (51.9)	0.942 (0.540–1.644)	0.834		
LA diameter	228	112 (48.7)	1.314 (0.947–1.824)	0.102		
LVEDVi	230	112 (48.7)	0.998 (0.984–1.012)	0.781		
LVEF	230	112 (48.7)	0.984 (0.958–1.011)	0.244		
LARS	216	104 (48.1)	0.968 (0.948–0.988)	**0**.**002**	0.993 (0.969–1.018)	0.595
LACI	230	112 (48.7)				
Tertile 1 vs. 2			0.928 (0.581–1.483)	0.754	0.911 (0.549–1.513)	0.719
Tertile 1 vs. 3			1.919 (1.251–2.943)	**0**.**003**	1.418 (0.839–2.396)	0.19

In a multivariate model including DBP but not SBP, the results remained unchanged HR = 1.017 95% CI = 0.999–1.036, *P* = 0.060.

AF, Atrial Fibrillation; CI, Confidence Interval; HR, Hazard Ratio; LA, left atrium; LACI, left atrioventricular coupling index; LARS, left atrial reservoir strain; LVEDVi, indexed left ventricular end-diastolic volume; LVEF, left ventricular ejection fraction.

## Discussion

In this brief report, the utility of LACI as a predictor of AF development in ESUS patients undergoing ILR implantation was explored. Although univariable analysis suggested that LACI is an effective predictor of future SCAF, after adjustment for age, this association was no longer significant, suggesting that the observed effect is largely explained by increasing age. Moreover, the log-rank assessments suggest that the only survival curves showing a significant difference were those examining the association of LACI tertiles with any duration of AF, rather than more prolonged 6 min or 5.5 h episodes.

LACI as a useful predictor of AF has been reported in a general population,^3^ hypertrophic cardiomyopathy,^6,7^ transthyretin amyloidosis,^8^ and post-AF ablation.^9^ However, none of these studies utilized ILR-detected AF, and several of the studies did not utilize age as a covariate, potentially explaining the different results in our study.

The association between increasing age and higher LACI has previously been described. Pezel et al. interrogated the Multi Ethnic Study of Atherosclerosis (MESA) cohort and demonstrated that age was independently associated with both increased LACI and an increased change in LACI over time.^10^ This association has been shown to be driven by increasing left atrial size with age, although the mechanism behind this remains unclear.

There are multiple advantages of LACI as a parameter. It is measurable across different modalities, with recent studies looking at the utility of MRI-measured LACI to prognosticate in heart failure.^4^ Moreover, the contributing parameters to LACI—indexed end-diastolic volumes of the left atrium and ventricle are a common part of minimum imaging datasets and are easily measured. Indeed, in our own dataset, of the 296 patients who underwent echocardiography, 230 (78%) had measurable LACI, comparable to other contemporaneous LA parameters, such as reservoir strain, with the majority of cases were LACI was not calculable predating the 2013 BSE minimum dataset.

In the ESUS cohort, transthoracic echocardiography is a key component of the initial workup, which makes echocardiographically derived LACI measurements particularly accessible as a marker in this cohort. This accessibility would allow for large-scale validation in either retrospective or prospective studies, and also underlines its possible utility as patients would not have to undergo further tests.

## Study limitations

The data for this study was derived from a single-centre retrospective analysis. Moreover, it is a relatively small population, especially when compared to other studies of predictors of AF. Nevertheless, it is the first study to look specifically at LACI as an AF predictor in the ESUS population, and utilize ILR-detected AF as the outcome measure.

## Conclusion

In this retrospective analysis, LACI was shown to be a useful univariable predictor of AF, but this association appears to be mediated by age. Given the ease with which LACI can be measured and its cross-modality measurability, larger studies in the ESUS population with ILR-detected AF are warranted to clarify whether LACI may have a useful role in clinical practice.

## Data Availability

The data are available from the corresponding author upon reasonable request.

## Lead author biography

Dr Rahul K. Chattopadhyay is a Clinical Research Fellow with the Vassiliou Group at the University of East Anglia, UK. His research interests include approaches to the prediction, detection and prevention of atrial fibrillation, especially in at-risk populations. Additionally, he is a Cardiology Registrar in the East of England, with an interest in heart failure and cardiac devices.
